# Detrimental Effect Elimination of Laser Frequency Instability in Brillouin Optical Time Domain Reflectometer by Using Self-Heterodyne Detection

**DOI:** 10.3390/s17030634

**Published:** 2017-03-20

**Authors:** Yongqian Li, Xiaojuan Li, Qi An, Lixin Zhang

**Affiliations:** Department of Electronics and Communication Engineering, North China Electric Power University, Baoding 071003, China; liyq@ncepubd.edu.cn (Y.L.); an-qi.122@163.com (Q.A.); zhanglxmail@126.com (L.Z.)

**Keywords:** Brillouin optical time domain reflectometer, laser frequency instability, Brillouin linewidth, self-heterodyne detection, Rayleigh scattering

## Abstract

A useful method for eliminating the detrimental effect of laser frequency instability on Brillouin signals by employing the self-heterodyne detection of Rayleigh and Brillouin scattering is presented. From the analysis of Brillouin scattering spectra from fibers with different lengths measured by heterodyne detection, the maximum usable pulse width immune to laser frequency instability is obtained to be about 4 µs in a self-heterodyne detection Brillouin optical time domain reflectometer (BOTDR) system using a broad-band laser with low frequency stability. Applying the self-heterodyne detection of Rayleigh and Brillouin scattering in BOTDR system, we successfully demonstrate that the detrimental effect of laser frequency instability on Brillouin signals can be eliminated effectively. Employing the broad-band laser modulated by a 130-ns wide pulse driven electro-optic modulator, the observed maximum errors in temperatures measured by the local heterodyne and self-heterodyne detection BOTDR systems are 7.9 °C and 1.2 °C, respectively.

## 1. Introduction

Due to the merits, such as high brightness and directivity, good monochromaticity, and coherence, lasers with high frequency and power stability have been widely used in the research of biology, medicine, chemistry, coherent optical communication and optical fiber sensing, wind LiDAR, and high-resolution spectroscopy [1,2,3,4,5,6,7]. Among these studies, optical fiber sensors based on Brillouin scattering have attracted much attenuation due to their capability to measure the distribution of temperature and/or strain, vibration and moisture in large civil structures, power cables, geological disasters, and road embankments [8,9,10,11,12,13]. In general, Brillouin-based distributed sensors can be implemented by using either stimulated Brillouin scattering (SBS) [4,12] or spontaneous Brillouin scattering (SPBS) [8,9,10]. Nevertheless, in a Brillouin optical time domain reflectometer (BOTDR) system, the round-trip delay time from launching the pulsed light to receiving the backscattered Brillouin light at the front end of a fiber can be used to determine the scattering location, and the frequency shift and intensity of spontaneous Brillouin light in the fiber can be used to simultaneously measure distributed temperature and strain by accessing only one end of fiber, offering extra advantages over the SBS-based Brillouin optical time domain analysis system.

To improve the efficiency of Brillouin scattering and extend the sensing distance of a BOTDR system, a laser with narrow-linewidth and high frequency stability is necessary for coherent detection of local reference and SPBS lights [10,14]. Geng et al. realized the distributed measurements of temperature and strain by using a fiber laser with 3-dB linewidth less than 10 kHz [15]. Hao et al. used a fiber laser with 4-kHz linewidth and a distributed feedback laser with 3-MHz linewidth to investigate the influence of laser linewidth on the system performance [16]. Unfortunately, the large coherent Rayleigh noise (CRN) and low SBS threshold seriously limited the system measurement accuracy due to the high coherence of ultra-narrow linewidth lasers. In order to reduce the CRN, improve the SBS threshold, and reduce the cost of the laser source, De Souza utilized a broad-band *Q*-switched erbium-doped fiber laser to reduce CRN and enhance the temperature resolution of a fiber distributed temperature sensor based on the Landau-Placzek ratio [17], and Soto et al. presented the use of multi-longitudinal mode Fabry-Perot (F-P) laser source and achieved the resolution of ~4.5 K in temperature and ~115 µε in strain at 25 km distance with a spatial resolution of 35 m [18]. By using lasers with different linewidths, the temperature and/or strain sensing have/has been realized in diverse BOTDR configurations. However, the influence of laser frequency instability on the performance of BOTDR system has received little attention. In a traditional local heterodyne detection BOTDR system, a time delay between the injection time of sensing pulse and the return time of backscattered Brillouin light from the scattering location in fiber is involved for the local reference light from laser, therefore the frequency variation induced by laser frequency instability during the delay time period may distort the Brillouin spectrum and cause a degradation of measurement accuracy with the increase of fiber length.

In this paper, a BOTDR system employing the self-heterodyne detection of Rayleigh and Brillouin scattering, which can effectively eliminate the detrimental effect of laser frequency instability on the temperature measurement accuracy, is proposed and demonstrated. The modeling analysis of Brillouin signals with laser frequency instability obtained by the local heterodyne and self-heterodyne detection is conducted, and two experimental setups for Brillouin spectrum measurements with different fiber lengths are used to determine the maximum usable pulse width immune to laser frequency instability in the self-heterodyne detection BOTDR system using broad- band laser. Finally, we compare the performance of BOTDR systems employing two lasers with different frequency stabilities based on the local heterodyne and self-heterodyne detection.

## 2. Modeling Analysis of Heterodyne Detection Brillouin Signals with Laser Frequency Instability

### 2.1. Local Heterodyne Detection

Brillouin scattering is continuously generated by the nonlinear interaction between injected optical photons and thermally excited acoustic phonons while an optical pulse propagates through an optical fiber. The returned Brillouin scattering with a round-trip time delay at the front end of fiber can be given by:

(1) $$E_{B}(t)=E_{S}(t)+E_{AS}(t)=\left\{\begin{array}{l} E_{S}\exp\left\{i\left\{2\pi \left[v_{0}(t_{0})-v_{B}(t_{0}+2nz/c)\right]t+\phi_{S}(t_{0}+2nz/c)\right\}\right\}+\\ E_{\mathrm{AS}}\exp\left\{i\left\{2\pi \left[v_{0}(t_{0})+v_{B}(t_{0}+2nz/c)\right]t+\phi_{\mathrm{AS}}(t_{0}+2nz/c)\right\}\right\} \end{array}\right\}$$

where *E*_S_ and *E*_AS_ are, respectively, the field intensities of backscattered Stokes and anti-Stokes lights, *t*_0_ is the initial moment that the pulsed light injects into the fiber, *v*_0_(*t*_0_) is the frequency of the fiber injected pulse light at moment *t*_0_, 2*nz*/*c* represents the time delay between the injection time of sensing pulse and the return time of backscattered Brillouin light from the scattering location *z* in the fiber, *n* is the refractive index of fiber core and *c* is the speed of light in vacuum, *v*_B_(*t*_0_ + 2*nz*/*c*) is the Brillouin frequency shift (BFS) of Brillouin light returned from the scattering location *z* in the fiber, and *φ*_S_(*t*_0_ + 2*nz*/*c*) and *φ*_AS_(*t*_0_ + 2*nz*/*c*) are, respectively, the phases of Stokes and anti-Stokes lights at the front end of fiber.

Provided that the output power of laser source is stable, the electrical field of local reference light from the laser source that meets and interferes with the returned Brillouin scattering light is expressed as:

(2) $$E_{L}(t)=E_{L}\exp\left\{i\left[2\pi v_{0}(t_{0}+2nz/c)t+\phi_{L}(t_{0}+2nz/c)\right]\right\}$$

where *E*_L_ is the field intensity of local reference light, *v*_0_(*t*_0_ + 2*nz*/*c*) and *φ*_L_(*t*_0_ + 2*nz*/*c*) are, respectively, the center frequency and the phase of local reference light at moment *t*_0_ + 2*nz*/*c.* Considering that *E*_S_ equals to *E*_AS_ for SPBS, the heterodyne detection signal of local reference and Stokes lights can be used to analyze the heterodyne detection signal of local reference and Brillouin lights. Assuming that the local reference and Stokes lights have aligned polarization, and then when the two lights are mixed up together and detected by a conventional photodetector (PD), the heterodyne detection signal can be obtained by:

(3) $$\begin{array}{cc} i(t) & =R\left[{\bar{E}}_{L}(t)+{\bar{E}}_{S}(t)\right]\left[E_{L}(t)+E_{S}(t)\right]\\ & =R\left\{P_{L}+P_{S}+2\sqrt{P_{L}P_{S}}\cos\left\{\begin{array}{l} 2\pi v_{B}(t_{0}+2nz/c)t+2\pi \left[v_{0}(t_{0}+2nz/c)-v_{0}(t_{0})\right]t+\\ \phi_{\mathrm{LS}}(t_{0}+2nz/c) \end{array}\right\}\right\} \end{array}$$

where *R* is the responsivity of the detector, ¯ represents the conjugate, *P*_L_ and *P*_S_ are, respectively, the powers of local reference and Stokes lights, *φ*_LS_(*t*_0_ + 2*nz*/*c*) that fluctuates within [0, 2π] following the uniform random distribution is the phase difference between local reference light and Stokes light generated at the location *z*. In Equation (3), the first two terms are direct current power terms, and the third term is the time-variant term carrying BFS and Brillouin intensity information. Assuming that the PD has a band-pass frequency characteristic, and then the obtained photocurrent corresponding to Brillouin signal can be expressed as:

(4) $$i_{\mathrm{LS}}(t)=2R\sqrt{P_{L}P_{S}}\cos\left\{2\pi v_{B}(t_{0}+2nz/c)t+2\pi \left[v_{0}(t_{0}+2nz/c)-v_{0}(t_{0})\right]t+\phi_{\mathrm{LS}}(t_{0}+2nz/c)\right\}$$

in which the term, *v*_0_(*t*_0_ + 2*nz*/*c*) − *v*_0_(*t*_0_), represents the frequency variation of laser source in the time interval of [*t*_0_, *t*_0_ + 2*nz*/*c*], and is related to the sensing distance or fiber length. From Reference [10] and Equation (4), the parameters of Brillouin signals at different scattering locations obtained by local heterodyne detection depend not only on the laser output frequency, fiber temperature and strain, but also on the frequency stability of laser source and the fiber length. Therefore, when the fiber keeps at constant temperature and strain, the fluctuations of local heterodyne detection Brillouin signals distributed along the fiber are caused not only by the thermal noise and shot noise of the detector, but also by the frequency variation of laser source during the delay time between the injection time of optical pulse and the meeting time of local reference light with the returned Brillouin scattering. To reduce the measurement errors caused by laser frequency instability, a method using self-heterodyne detection of Rayleigh and Brillouin scattering lights is proposed to obtain the temperature and strain information along the fiber accurately.

### 2.2. Self-Heterodyne Detection of Rayleigh and Brillouin Scattering

When an optical pulse propagates in fiber, Rayleigh and Brillouin scattering lights generated at the scattering location *z* are with the same characteristics of laser frequency, and the electrical field of returned Rayleigh scattering signal at the front end of fiber can be given by:

(5) $$E_{R}(t)=E_{R}\exp\left\{i\left[2\pi v_{R}(t_{0}+2nz/c)t+\phi_{R}(t_{0}+2nz/c)\right]\right\}$$

where *E*_R_ is the field intensity of Rayleigh scattering light, *v*_R_(*t*_0_ + 2*nz*/*c*) and *φ*_R_(*t*_0_ + 2*nz*/*c*) are the frequency and phase of Rayleigh scattering light from the location *z*, respectively. Being unaffected by the fiber loss, temperature, and strain, the Rayleigh frequency *v*_R_(*t*_0_ + 2*nz*/*c*) along the entire fiber keeps equal to *v*_0_(*t*_0_) in the same input pulse period. Considering that *E*_S_ equals to *E*_AS_ for SPBS, the self-heterodyne detection signal of Rayleigh and Stokes lights can be used to analyze the heterodyne detection signal of Rayleigh and Brillouin lights. Since Rayleigh and Stokes lights occurred at the same location in fiber have aligned polarization, when the two lights are detected by a PD, the self-heterodyne detection signal can be expressed as:

(6) $$\begin{array}{cc} i(t) & =R\left[{\bar{E}}_{R}(t)+{\bar{E}}_{S}(t)\right]\left[E_{R}(t)+E_{S}(t)\right]\\ & =R\left\{P_{R}+P_{S}+2\sqrt{P_{R}P_{S}}\cos\left[2\pi v_{B}(t_{0}+2nz/c)t+\phi_{\mathrm{RS}}(t_{0}+2nz/c)\right]\right\} \end{array}$$

in which *P*_R_ is the power of Rayleigh scattering light, *φ*_RS_(*t*_0_ + 2*nz*/*c*) that fluctuates within [0, 2π] following the uniform random distribution is the phase difference between Rayleigh and Stokes lights at the front end of fiber. After the direct current terms in Equation (6) are filtered by a band-pass filter, the self-heterodyne detection signal can be obtained as:

(7) $$i_{\mathrm{RS}}(t)=2R\sqrt{P_{R}P_{S}}\cos\left[2\pi v_{B}(t_{0}+2nz/c)t+\phi_{\mathrm{RS}}(t_{0}+2nz/c)\right]$$

According to Equation (7), Brillouin signal obtained by the self-heterodyne detection of Rayleigh and Brillouin scattering at the location *z* depends only on the laser output frequency, fiber temperature and strain, and is not influenced by the frequency instability of laser source. However, in an actual BOTDR sensing system, there are lots of scattering points within a single spatial resolution of *δz* = *cW*/(2*n*) that corresponds to the input optical pulse width *W*, thus, the obtained Brillouin signal by self-heterodyne detection contains all beat signals of Brillouin scattering light generated at any location with Rayleigh scattering lights generated at all locations within the fiber length of a spatial resolution, so that the self-heterodyne detection Brillouin spectra are affected by the laser frequency variation during the pulse width. Therefore, the influences of laser frequency instability on the self-heterodyne detection Brillouin spectra with different spatial resolutions that is equivalent to different fiber lengths need to be studied, from which the maximum usable pulse width immune to laser frequency instability for a broad-band laser with low frequency stability can be obtained.

## 3. Influence of Laser Frequency Instability on Heterodyne Detection Brillouin Spectra

**Figure 1:**
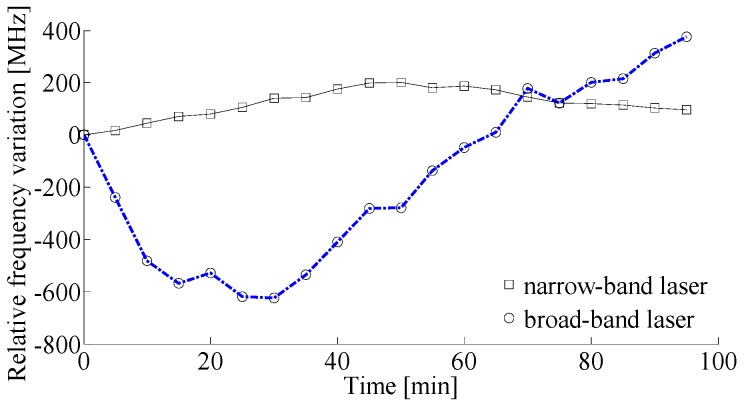
Relative frequency variations of the two laser sources.

In order to study the influence of laser frequency instability on the system performance, two distributed feedback semiconductor laser diode (DFB-LD) components with different stabilities, one narrow-band laser (TL-2020-C-102A, Santur CO., Ltd., Fremont, CA, USA) with a 3-dB linewidth of 1.86 MHz and a center wavelength of 1550.12 nm, and another broad-band laser (OST-DFB-1550-SM, Fby Photoelectric Technology CO., Ltd., Shenzhen, China) with a 3-dB linewidth of 55.23 MHz and a center wavelength of 1550.08 nm were used. The frequency variations of the two lasers relative to their own initial frequencies in about 95 min were measured every 5 min by an optical spectrum analyzer with a resolution bandwidth of 26 MHz and shown in Figure 1. It can be seen clearly from Figure 1 that the frequency variation of the narrow-band laser is approximately 201.6 MHz, and the frequency variation of the broad-band laser is nearly 1 GHz, thus, the frequency stability of the broad-band laser is much lower than that of the narrow-band laser.

**Figure 2:**
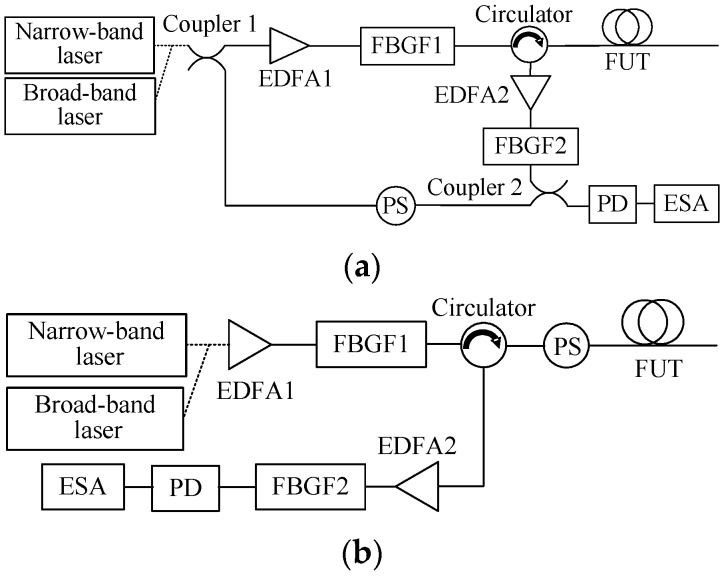
Experimental setups for Brillouin spectrum measurement employing narrow-band and broad-band lasers based on (**a**) local heterodyne detection and (**b**) self-heterodyne detection.

To clarify the influence of laser frequency instability on Brillouin scattering spectra with different spatial resolutions, two experimental setups for Brillouin spectrum measurement based on local heterodyne and self-heterodyne detection with the narrow-band and broad-band lasers are constructed, as shown in Figure 2. The output continuous lights of the two lasers were amplified by erbium doped fiber amplifier 1 (EDFA1) with a gain of 45 dB, then launched into the fiber under test (FUT) to generate SPBS and Rayleigh scattering signals after having filtered out the amplified spontaneous emission (ASE) noise by fiber Bragg grating filter 1 (FBGF1) with a center wavelength of 1549.969 nm and a 3-dB bandwidth of 0.346 nm. The backscattered lights were amplified by EDFA2 with a gain of 35 dB, and the ASE noise from EDFA2 and anti-Stokes light from the fiber were filtered out by FBGF2 with a center wavelength of 1550.151 nm and a bandwidth of 0.25 nm. Stokes lights generated from the fibers with different lengths that correspond to different spatial resolutions were mixed up together with local reference lights and Rayleigh lights in a PD with a 11.9-GHz bandwidth for local heterodyne detection and self-heterodyne detection, respectively, and the heterodyne detection Brillouin spectra are measured by an electrical spectrum analyzer (ESA) with a resolution bandwidth of 8 MHz. To reduce polarization-induced fading noise, a polarization scrambler (PS) was used in the local reference light path in Figure 2a or at the front end of the fiber in Figure 2b.

**Figure 3:**
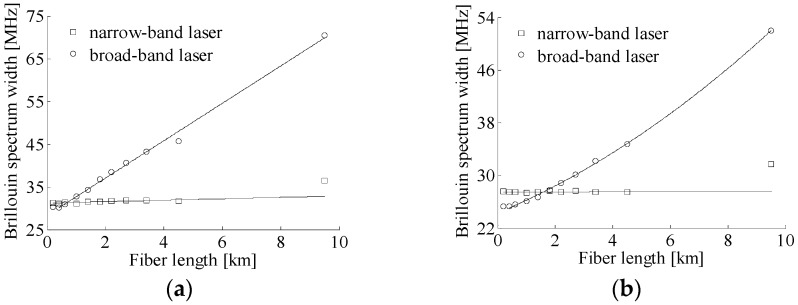
Brillouin spectrum width versus fiber length with different lasers: (**a**) local heterodyne detection and (**b**) self-heterodyne detection.

The test fibers are standard single-mode fibers with each end being angle polished to avoid the undesired Fresnel reflection at the connectors, and the fiber lengths used in measuring Brillouin spectra are given in Table 1. The power of local reference light is set to be about 814.9 µW, giving a PD input power of 394.3 µW, and remains constant for the test fibers with different lengths in the local heterodyne detection system, and the PD input powers are approximately equal to 177.6 µW for all test fibers in the self-heterodyne detection system. In the heterodyne detection systems with different lasers, the Brillouin spectrum widths are obtained by fitting the Brillouin spectra under different fiber lengths measured by the two setups for three times with a Lorentzian curve, and the averaged Brillouin spectrum width versus fiber length is depicted in Figure 3.

**Figure 4:**
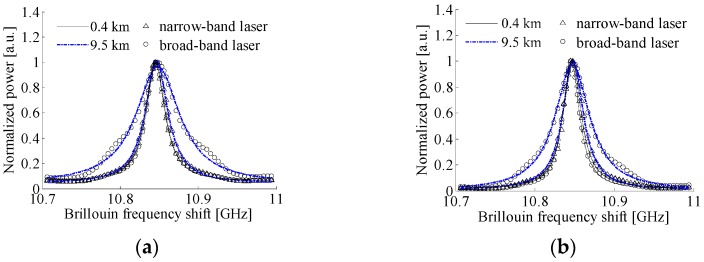
Brillouin spectra of 0.4 km and 9.5 km long fibers with different lasers: (**a**) local heterodyne detection and (**b**) self-heterodyne detection.

In Figure 3, it is seen that, with the increase of fiber length, Brillouin spectrum widths detected by the two systems remain first almost constant, then increase slowly when employing the narrow- band laser with high frequency stability. From the Brillouin spectra of 0.4 km and 9.5 km long fibers shown in Figure 4 measured by the two systems using different lasers, the Brillouin spectra with the narrow-band laser obey the Lorentzian distribution, and the fitted Brillouin spectrum widths are, respectively, 31.1 MHz, 27.5 MHz, 36.4 MHz, and 31.7 MHz, which indicates that the influence of laser frequency instability on Brillouin spectra is relatively small in the two systems when using a narrow-band laser. For the laser source with low frequency stability, however, the returned Brillouin spectrum is superposed by Brillouin spectra from different locations with different center frequencies and different round trip delay times, which may result in the broadening and distortion of the heterodyne detection Brillouin spectra. As shown in Figure 3a, Brillouin spectrum width measured by the local heterodyne detection broadens rapidly in a linear relation with the increase of fiber length longer than 0.4 km. Compared to the Brillouin spectrum of 0.4 km long fiber that obeys Lorentzian distribution and has a spectrum width of 30.2 MHz, the Brillouin spectrum of 9.5 km long fiber shown in Figure 4a manifests serious distortion and the fitted spectrum width with a Lorentzian curve is 70.5 MHz; therefore, the influence of laser frequency instability in the local heterodyne detection system is inevitable and non-negligible.

In the self-heterodyne detection system, since Rayleigh and Brillouin lights scattered from the same location have uniform characteristics of laser frequency, the effect of laser frequency instability on Brillouin signal can be eliminated as described in Equation (7). However, for the broad-band laser based system, Rayleigh and Brillouin lights from different locations are affected by laser frequency instability and interfere between different components, thereby resulting in the Brillouin spectrum broadening rapidly in a nearly quadratic polynomial relation with the increase of fiber length longer than 0.4 km, as shown in Figure 3b. As depicted in Figure 4b, the self-heterodyne detection Brillouin spectrum of 9.5 km long fiber also presents a relatively small distortion and has a fitted spectrum width of 51.93 MHz. It is interesting to note that when the fiber length is shorter than 0.4 km, the Brillouin spectra measured by the two systems using broad-band laser obey the Lorentzian distribution and the fitted spectrum width almost keeps unchanged with the increase of fiber length, and has a value smaller than that from the narrow-band laser based systems as shown in Figure 3, which indicates that the detrimental effect of laser frequency instability on system performance can be effectively eliminated for the input pulse width narrower than 4 µs in the BOTDR system with the broad-band laser based on self-heterodyne detection of Rayleigh and Brillouin scattering, and for the local heterodyne detection based system using a sensing fiber not longer than 0.4 km.

It should be mentioned that due to the distribution of inversed population at different energy band in the active layer of DFB-LD, the laser output is composed of frequency discrete and amplitude time-variant spectral lines in a single longitudinal mode spectrum [19]. According to reference [20], the heterodyne detection Brillouin spectrum with broad-band laser composed of independent ultra-narrow spectral lines is the superposed Brillouin spectrum from different spectral lines, and since the dependence of BFS on probe light wavelength is only 7 MHz/nm around 1550 nm, the Brillouin spectrum width depends mainly on the linewidth of a single spectral line. Therefore, the heterodyne detection Brillouin spectrum widths shown in Figure 3 are narrower than those when employing the narrow-band laser for short fibers.

## 4. Experiment and Discussion

To demonstrate the detrimental effect elimination of laser frequency instability on Brillouin signals experimentally by the self-heterodyne detection of Rayleigh and Brillouin scattering, the experimental setups based on local heterodyne detection and self-heterodyne detection BOTDR techniques are constructed.

### 4.1. Local Heterodyne Detection BOTDR System

**Figure 5:**
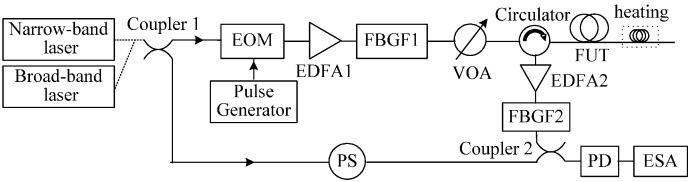
Experimental setup of temperature sensing based on the local heterodyne detection BOTDR technique.

The experimental setup of temperature sensing based on local heterodyne detection BOTDR technique is shown in Figure 5. The output lights of the narrow-band and broad-band lasers were divided into two beams, one beam was modulated to optical pulse with a width of 130 ns and a peak power of 27.8 dBm by an electro-optic modulator (EOM) as probe beam, and the other beam was used as a local reference light. The optical pulse was amplified by EDFA1 with a gain of 35 dB, filtered by FBGF1, and adjusted by a variable optical attenuator (VOA), and then launched into the FUT through an optical circulator to generate SPBS light. The Brillouin light was amplified by EDFA2, and the ASE noise and anti-Stokes light from EDFA2 output were filtered out by FBGF2. The filtered backscattered Stokes light with a round trip delay time of 2*nz*/*c* beats with local reference light in a PD with 11.9 GHz bandwidth, and the PD input power was about 403 µW.

**Figure 6:**
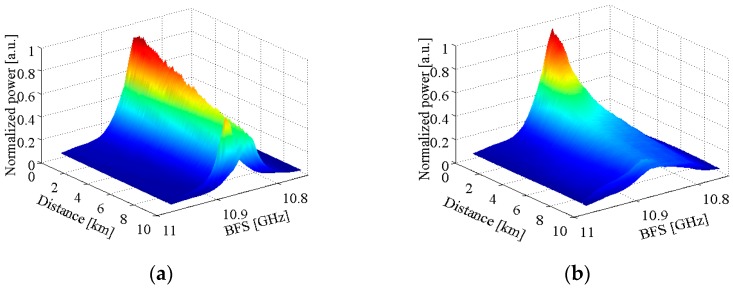
3D power spectra of the local heterodyne detection Brillouin signals with (**a**) narrow-band laser and (**b**) broad-band laser. The temperature of the thermostatic water bath is set at 50 °C.

**Figure 7:**
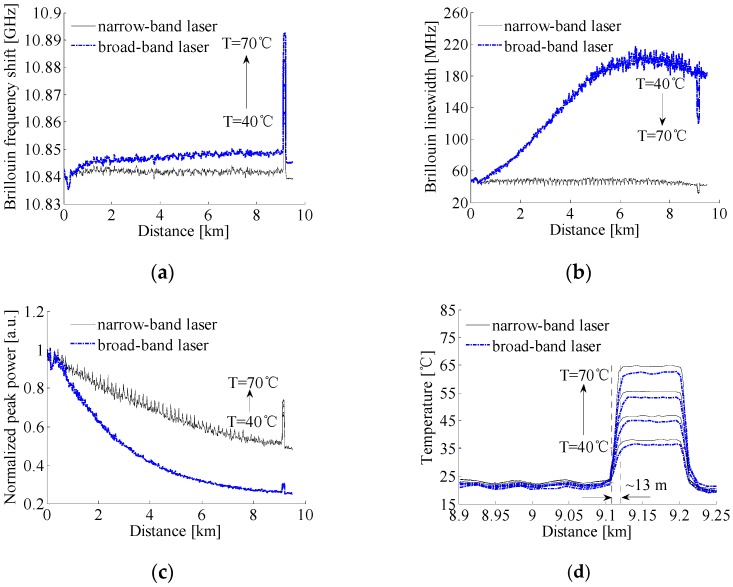
Distribution of Brillouin signals and demodulated temperature along the fiber obtained by local heterodyne detection system: (**a**) Brillouin frequency shift; (**b**) Brillouin linewidth; (**c**) Brillouin peak power; and (**d**) temperature.

In Figure 5, a 9.5 km long single-mode fiber was used as sensing fiber and the measurements were performed at room temperature of 21.7 °C. The 100 m long fiber near the far end was wound with no tension to avoid any strain and heated to a temperature of 40 °C–70 °C by a thermostatic water bath with a temperature accuracy of ±0.01 °C. The ESA with a resolution bandwidth of 8 MHz operated in the “zero-span” mode was used to acquire the power traces along the sensing fiber at different beat frequencies. The beat frequency was adjusted from 10.7515 GHz to 10.9835 GHz by a step of 8 MHz and each trace was averaged for 5000 times. The obtained 3D power spectra of the local heterodyne detection Brillouin signals with narrow-band and broad-band lasers are shown in Figure 6. The spectrum parameters are obtained by fitting the measured spectra with a Lorentzian curve, and the comparison of all spectrum parameters and demodulated temperatures is made between the narrow-band laser and broad-band laser measurements, as shown in Figure 7. For convenience of comparison, we normalized the peak power traces obtained from the systems with different lasers.

In the local heterodyne detection system, the measured Brillouin signals from different scattering locations depend not only on the laser output frequency, fiber temperature, and strain, but also on the frequency variation of laser source and the distances from input end to scattering locations of the fiber. As shown in Figure 7a, when the fiber sensing distance is short, the detrimental effect of laser frequency instability on BFS is small, and the measured BFSs are approximately equal in use of the two lasers. Compared to the system employing the narrow-band laser with high frequency stability, with the increase of sensing distance, the frequency instability of laser source results in the BFS along the unheated fiber increasing slowly to an averaged value of 10.8487 GHz when employing the broad-band laser. The averaged BFSs and averaged temperatures demodulated from the BFSs by the calibrated fiber temperature coefficient of 1.07 MHz/°C for the 100 m heated fiber placed in the thermostatic water bath at different temperatures in the range of 40 °C–70 °C are given in Table 2. From Table 2, the maximum temperature measurement error is 7.9 °C among the four temperatures. As shown in Figure 7b, since the broad-band laser with low frequency stability is composed of independent and ultra-narrow spectral lines, the measured Brillouin linewidths with broad-band laser near the fiber initial end are almost the same as that using the narrow-band laser, and almost remain constant for the fiber distance shorter than 0.4 km, which is well in accordance with the results in Section 3. Due to the frequency instability of the broad-band laser, the coherence between the returned Brillouin scattering from the location *z* and the local reference light meeting with the returned Brillouin scattering at moment *t*_0_ + 2*nz*/*c* becomes worse, resulting in the Brillouin linewidth increasing quickly to a constant before slowly decreasing, with the increase of sensing distance. Meanwhile, the normalized Brillouin peak power along the fiber presents a trend of rapid decline to a relatively small value as shown in Figure 7c, which causes the large measurement error of 7.9 °C.

As shown in Figure 7a–c measured by the system with narrow-band laser, BFSs fluctuate from 10.8352 GHz to 10.8442 GHz around an averaged BFS value of 10.842 GHz, Brillouin spectrum widths almost remain constant and the normalized Brillouin peak power decreases exponentially along the unheated fiber. From the averaged BFSs and averaged temperatures for the heated fiber given in Table 2, the maximum measurement error is 5.3 °C for different thermostatic water bath temperatures. From Figure 7d, the system spatial resolutions obtained by calculating the 10% to 90% response distance are both to be about 13 m for the two lasers. It is worthy to be mentioned that the abrupt increase of BFSs, decrease of Brillouin spectrum widths and increase of normalized peak powers for the heated fiber with broad-band and narrow-band lasers shown in Figure 7a–c are accorded well with the previously reported results by Nikles et al. [21] and/or Soto et al. [18] in different systems and/or by different lasers. It is concluded that the laser source with high frequency stability can overcome the disadvantages of a broad-band laser caused by the low frequency stability, but the achieved measurement error of 5.3 °C is still far from the requirement for accurate distributed temperature information in real applications.

### 4.2. Self-Heterodyne Detection BOTDR System

**Figure 8:**
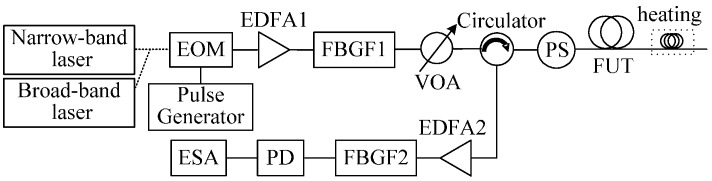
Experimental setup of temperature sensing based on the self-heterodyne detection BOTDR technique.

In order to eliminate the detrimental effect of laser frequency instability on Brillouin signals in the local heterodyne detection system, a useful method for measuring Brillouin signals by the self-heterodyne detection of Rayleigh and Brillouin scattering lights is proposed, and the BOTDR experimental setup based on the proposed method is shown in Figure 8. The output lights of the narrow-band and broad-band lasers were modulated by EOM to optical pulse with a width of 130 ns and a peak power of 27.8 dBm as probe beam. The optical pulse was passed through the EDFA1 and FBGF1, then launched into the FUT through an optical circulator to generate Rayleigh scattering and SPBS lights. The two weak lights were amplified by EDFA2, and the ASE noise and anti-Stokes light were filtered out by FBGF2. The Stokes light beats with Rayleigh light in a PD with a 11.9-GHz bandwidth, and the PD input power was set at about 402.7 µW. To reduce the polarization-induced fading noise and increase the SBS threshold limited fiber input power, a PS was used at the front end of the fiber.

**Figure 9:**
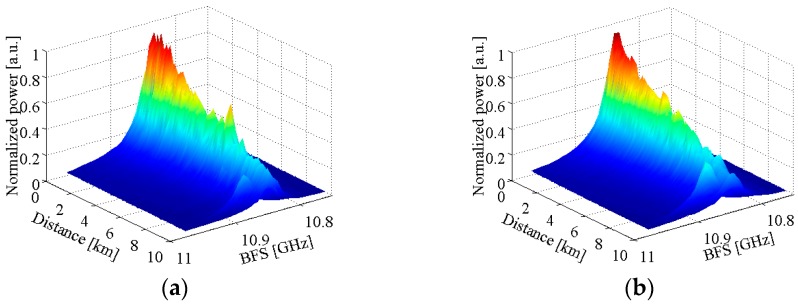
3D power spectra of the self-heterodyne detection Brillouin signals with a (**a**) narrow-band laser and (**b**) broad-band laser. The temperature of the thermostatic water bath is set at 50 °C.

**Figure 10:**
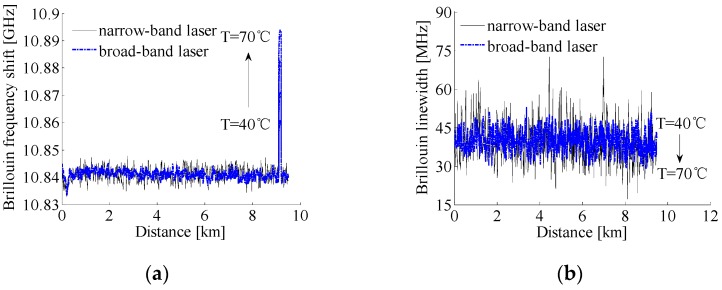

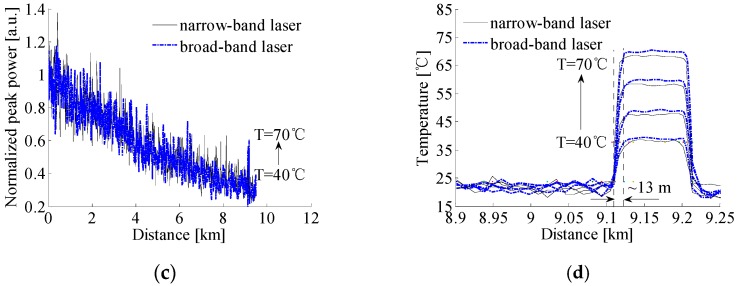
Distribution of Brillouin signals and demodulated temperature along the fiber based on the self-heterodyne detection: (**a**) Brillouin frequency shift; (**b**) Brillouin linewidth; (**c**) Brillouin peak power; and (**d**) temperature.

Again, the ESA with a 8-MHz resolution bandwidth operated in the “zero-span” mode was used to acquire the power traces along the sensing fiber at different beat frequencies. The beat frequency was adjusted from 10.7529 GHz to 10.9849 GHz with a 8-MHz step and each trace was averaged for 5000 times, and the obtained 3D power spectra of self-heterodyne detection Brillouin signals with the two lasers are depicted in Figure 9. The spectrum parameters are obtained by fitting the measured spectra with a Lorentzian curve, and the comparison of all spectrum parameters and demodulated temperatures is made between the narrow-band and broad-band laser measurements, as shown in Figure 10. For convenience of comparison, we normalized the peak power traces obtained from the system with different lasers.

From Figure 10a–c, it is clearly seen that the parameters of Brillouin signals along the fiber obtained by the setup using a broad-band laser are in good accordance with those using a narrow- band laser, which are much different from the results in Figure 7 obtained by the setup given in Figure 5. Since the Rayleigh and Brillouin scattering from the same scattering location are with the same characteristics of laser frequency, aligned polarization, and are highly coherent, the detrimental effect of laser frequency instability on the measured Brillouin signals is eliminated by the self-heterodyne detection. Although the averaged BFSs of the unheated fiber measured by the systems employing the narrow-band and broad-band lasers are both about 10.8411 GHz, the root mean square (RMS) error of the BFS with the narrow-band laser is 2.5 MHz that is much larger than the achieved RMS error of 1.5 MHz with the broad-band laser due to the much larger CRN. As a result, the achieved maximum measurement errors among four averaged temperatures demodulated from the averaged BFSs for the 100 m heated fiber at different thermostatic water bath temperatures are 2.4 °C and 1.2 °C, respectively, when using the narrow-band and broad-band lasers, as is given in Table 3.

As shown in Figure 10b, the greater volatility of the fitted Brillouin linewidths observed for the narrow-band laser also comes, mainly, from the greater amplitude fluctuations of the Rayleigh scattering signal induced by the larger CRN, and the RMS errors of Brillouin linewidths for the unheated fiber with the narrow-band and broad-band lasers are 7.6 MHz and 4.3 MHz, respectively. As the Rayleigh and Brillouin signals generated from a single spatial resolution are with the similar features of laser frequency, polarization and coherence, the spectral broadening due to the frequency instability of laser source is eliminated effectively by employing the self-heterodyne detection of Rayleigh and Brillouin scattering, so that the degradation of measurement accuracy at the fiber end can be avoided. Meanwhile, the normalized Brillouin peak powers along the unheated fiber obtained by the two different lasers decrease together exponentially with the increase of sensing distance, and the RMS errors of them are 0.096 and 0.071, respectively. As shown in Figure 10d, the system spatial resolutions obtained by calculating the 10% to 90% response distance are both about 13 m for the two lasers. On the basis of the above results, in the systems based on the self-heterodyne detection of Rayleigh and Brillouin scattering, thanks to the greatly-reduced CRN by using the broad-band laser, the RMS errors of Brillouin signals and the measurement error of temperatures have been reduced effectively.

Compared to the local heterodyne detection system with narrow-band laser, the fluctuations of BFSs, linewidths and peak powers along the fiber measured by the self-heterodyne detection of Rayleigh and Brillouin scattering becomes larger as a result of the existence of coherent Rayleigh noise in the self-heterodyne detection BOTDR system, which can be reduced by using a multi-wavelength laser source or a multi-longitudinal mode F-P laser source.

## 5. Conclusions

A BOTDR sensor system for eliminating the detrimental effect of laser frequency instability on Brillouin signals, based on the self-heterodyne detection of Rayleigh and Brillouin scattering, has been proposed and demonstrated. We analyzed and compared the Brillouin scattering spectra from fibers with different lengths obtained by the local heterodyne and self-heterodyne detection systems employing two lasers with different frequency stabilities, and the results show that the detrimental effect of laser frequency instability on the system performance can be effectively eliminated for the pulse widths narrower than 4 μs in the self-heterodyne detection BOTDR system using a broad-band laser. The BOTDR systems based on the local heterodyne and self-heterodyne detection employing different lasers are constructed. The experiments using the self-heterodyne detection BOTDR system have demonstrated the feasibility of the proposed method. Compared with the observed maximum temperature errors of 5.3 °C and 7.9 °C measured by the local heterodyne detection with the narrow-band and broad-band lasers, the maximum temperature errors obtained by the narrow-band and broad-band laser measurements based on the self- heterodyne detection are reduced to 2.4 °C and 1.2 °C, respectively. The results show that the temperature accuracy is improved effectively by eliminating the influence of laser frequency instability and reducing the CRN by the use of self-heterodyne detection and broad-band laser in BOTDR system, and the spatial resolution and measurable range could in principle be further improved.

## Figures and Tables

**Table 1:** Fiber lengths used in Brillouin spectrum measurement.

Fiber length [km]	0.2	0.4	0.6	1.0	1.4	1.8
	2.2	2.7	3.4	4.5	9.5	

**Table 2:** Measured averaged BFS and demodulated temperature of the heated fiber at different thermostatic water bath temperatures.

Temperature [°C]	Measured Averaged BFS [GHz]		Demodulated Temperature [°C]	
	Narrow-Band Laser	Broad-Band Laser	Narrow-Band Laser	Broad-Band Laser
40	10.8594	10.8643	38.0	36.3
50	10.8686	10.8733	46.6	44.7
60	10.8782	10.8825	55.5	53.3
70	10.8880	10.8919	64.7	62.1

**Table 3:** Measured averaged BFS and demodulated temperature of the heated fiber at different thermostatic water bath temperatures.

Temperature [°C]	Measured Averaged BFS [GHz]		Demodulated Temperature [°C]	
	Narrow-Band Laser	Broad-Band Laser	Narrow-Band Laser	Broad-Band Laser
40	10.8585	10.8596	38.0	39.0
50	10.8688	10.8701	47.6	48.8
60	10.8801	10.8818	58.1	59.7
70	10.8909	10.8927	68.2	69.9
